# Molecular Basis of the Differentiation and Function of Virus Specific Follicular Helper CD4^+^ T Cells

**DOI:** 10.3389/fimmu.2019.00249

**Published:** 2019-02-15

**Authors:** Qizhao Huang, Jianjun Hu, Jianfang Tang, Lifan Xu, Lilin Ye

**Affiliations:** ^1^Cancer Center, The General Hospital of Western Theater Command, Chengdu, China; ^2^Institute of Immunology, Third Military Medical University, Chongqing, China

**Keywords:** follicular T helper cells, acute viral infection, chronic viral infection, differentiation, CD4 T cells +

## Abstract

During viral infection, virus-specific follicular helper T cells provide important help to cognate B cells for their survival, consecutive proliferation and mutation and eventual differentiation into memory B cells and antibody-secreting plasma cells. Similar to Tfh cells generated in other conditions, the differentiation of virus-specific Tfh cells can also be characterized as a process involved multiple factors and stages, however, which also exhibits distinct features. Here, we mainly focus on the current understanding of Tfh fate commitment, functional maturation, lineage maintenance and memory transition and formation in the context of viral infection.

## Introduction

Based on the biological process, viral infections can be divided into two groups: acute viral infection and chronic viral infection. During acute infections, virus is thoroughly eliminated by the orchestration between both innate and adaptive immune cells; whereas, certain types of viruses can effectively evade immune system and persist at a certain level in the host for long time in chronic infections (1). Numbers of specific immune effector mechanisms, coordinating with non-specific defense mechanisms, prevent or eliminate most viral infections. In terms of adaptive immune cells, CD8^+^ T cell- and CD4^+^ T cell-mediated immune responses play a critical role in the control of viral infection. During acute viral infection, virus-specific CD8^+^T cells differentiate into cytotoxic T lymphocytes (CTL) to efficiently eliminate virus-infected target cells and progressively transit into memory CD8^+^ T cells after viral eradication. Memory CD8^+^ T cells are maintained for a long time in the absence of antigen and can exert rapid effector functions in response to previously encountered antigens.

After a transit time in the blood, the majority of mature naïve CD4^+^ T cells produced by the thymus migrate to secondary lymphoid tissues, continually patrolling, and browsing for antigens they can recognize. After entering a lymph node, T cells scan the processed peptide-MHC complexes on the surface of DCs in the paracortex or T-cell zone. DCs that have processed antigen at the sites of infection arrive in the paracortex soon after infection. Upon viral infection, virus-specific CD4^+^T cells mainly differentiate into Th1 and Tfh (follicular helper T cell) cells, but not other helper subsets, such as Th2, Th17, and Th9 due to the strong type-I inflammation. And the divergence of Tfh and Th1 differentiation fates begin immediately after activation and are faithfully maintained through the life cycle (2). Through interactions between S1P1 receptors and S1P, the Th1 subset leaves the lymph node and travel to sites of infection. And they predominantly function through secreting IL-2, IFN-γ and TNFα and are responsible for many typical cell-mediated effects, including activation of CTL and macrophages. In contrast, virus-specific Tfh cells, characterized by high expression of chemokine receptor CXCR5, are endowed with the ability of migrating into B cell follicles in response to chemokine CXCL13 (3, 4), where they facilitate the maturation of GC B cells by interacting with cognate virus-specific B cells and providing “help” signals such as interleukin 21 (IL-21), IL-4, CD40L and inducible costimulatory molecules (ICOS) (5).

**Figure 1:**
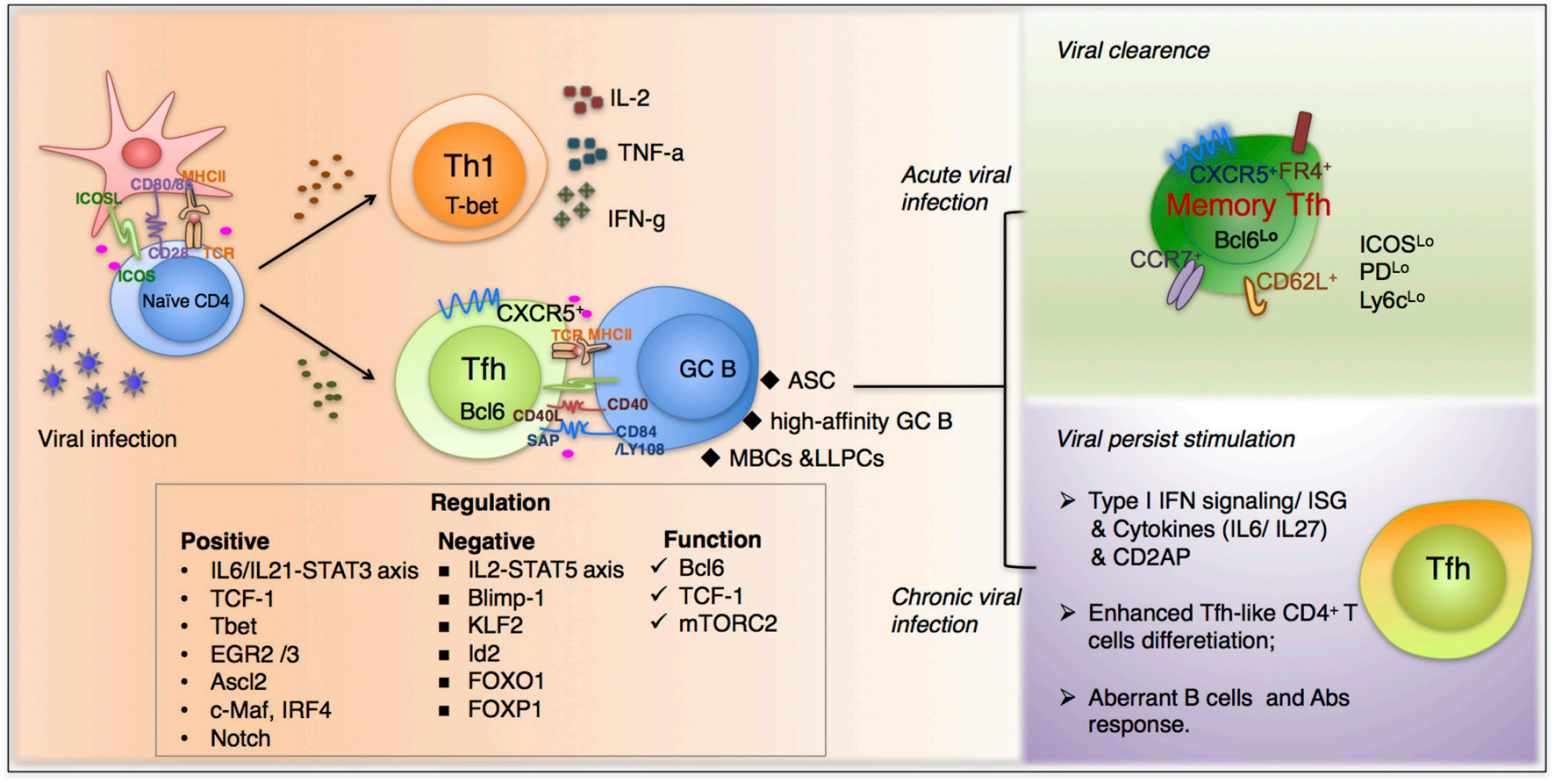
The fate commitment of Tfh cells during viral infection. Upon viral infection, virus-specific CD4^+^T cells mainly differentiate into Th1 and Tfh cells. Th1 subset predominantly function through secreting IL-2, IFN-γ, and TNFα and is responsible for many typical cell-mediated effects. In contrast, virus-specific Tfh cells, characterized by high expression of chemokine receptor CXCR5, are endowed with the ability of migrating into B cell follicles in response to chemokine CXCL13. The fate commitment and function of Tfh cells require fine-tuned cooperation of cognate p-MHCII molecular interactions, co-stimulation, together with polarizing cytokine signals and other factors. Moreover, upon viral clearance, a proportion of Tfh cells will differentiation in memory cells, which are more active when encountered with the same pathogens. Additionally, during chronic viral infections, Tfh cells accumulate gradually and exhibit a distinguished transcriptional profile compared with that in acute infections. And many factors participate in the unique differentiation pattern during persistent infection, including type I interferon signaling, cytokines from IL-6 family et al.

Tfh differentiation is generally characterized as a multistage, multifactorial process (Figure 1) (6). Upon recognition of virus peptide-MHC complex (p-MHC) presented by dendritic cells (DCs), CD4^+^ T cells adopting Tfh fate upregulate the “master regulator” Bcl-6 (7–9) within 2 or 3 days (10, 11). After engagement with DCs, Tfh cells move to the T-B border by upregulating CXCR5 and down-regulating CCR7 (10, 12). Here, they interact with cognate B cells and get sufficient signals that further support them migrating into B cell follicles and initiating GC reactions (13). During this process, the expression of Bcl-6 is enhanced, propelling the maturation of fully functional Tfh cells (14). Contrarily, Blimp1 (B lymphocyte-induced maturation protein-1), mainly expressed by non-Tfh effector cells, inhibits the expression of Bcl-6 and negatively regulates Tfh cell differentiation (7). Although the majority of Tfh cells originated from precursors in lymphoid tissues, several groups confirmed the existence of circulating CXCR5^+^ CD4^+^ T cells in mice or humans with ongoing immune responses, which were termed as peripheral Tfh (pTfh) (15–19). For instance, He et al. (18) demonstrated that pTfh consist of two parts: “effector” pTfh and “resting” pTfh cells, identified as CCR7^lo^PD-1^hi^ and CCR7^hi^PD-1^lo^, respectively. They found that CCR7^lo^PD-1^hi^CXCR5^+^ CD4^+^ T cells express large amounts of IL-21, a key cytokine secreted by Tfh cells to support GC responses. And this population is able to further differentiate into mature Tfh cells and initiate GC formation. They believed that CCR7^lo^PD-1^hi^ Tfh precursor cells can circulate to non-draining secondary lymphoid organs and rapidly differentiate into mature Tfh cells to support fast GC formation upon antigen reencounter. However, the underlying mechanisms of the ontology and differentiation of this population remain unsolved.

Tfh cells are essential for antibody-mediated humoral immunity against various pathogens. This review primarily focuses on the current understanding of the fate commitment, functional maturation, and memory formation of Tfh cells during acute viral infection. Moreover, we also focus on the role of Tfh cells during chronic viral infection, especially in HIV infection. Finally, we discuss the potential in boosting viral-specific Tfh cells for improving efficacies of anti-viral vaccines.

## The Fate Commitment of Virus-Specific Tfh Cells vs. Th1 Cells

The fine-tuned cooperation of cognate p-MHCII molecular interactions, co-stimulation, together with polarizing cytokine signals initiate the differentiation of functionally divergent CD4^+^ T helper (Th) cell subsets from their precursors (20). Of note, during acute viral infection, commitment to the Tfh lineage vs. Th1 lineage emerges as early as 24 to 48 h after infection. The dichotomous commitment of Tfh cells vs. Th1 cells is largely linked to reciprocal regulation between key transcription factors Bcl6 and T-bet, and Bcl6 and Blimp-1 (21, 22).

At the priming stage, DCs regulate Tfh cell differentiation by controlling Clec9A expression, which facilitates the formation of a long-term immune synapse between DCs and T cells to promote Tfh differentiation (23, 24). Indeed, published work (25–27) has found that 24 h after T cell activation, T cells carrying high affinity TCRs can form long dynamic immune synapses with DC and are more inclined to differentiate into Tfh but not Th1 cells. In addition to interactions between membrane proteins of APC and Tfh precursors, secreted cytokines interleukin-6 (IL-6) and IL-21 also contribute to Tfh differentiation. Several groups confirmed that IL-6 and IL-21 signaling via the transcription factor STAT3 enhances the upregulated expression of Bcl6, which is the master regulator of Tfh differentiation. Nonetheless, IL-2 suppresses Tfh fates by activating STAT5 and restricting STAT3 binding to the *Bcl6* locus and also by promoting the expression of Blimp-1, which divert differentiation away from the Tfh pathway (20, 28–31). Propelled by the antagonism of Bcl6 and Blimp-1, activated CD4^+^ T cells undergo a bimodal fate decision during acute viral infection: becoming either Tfh (Bcl6^+^Blimp1^−^) cells or Th1 (Bcl6^−^Blimp1^+^) cells. Notably, the transcription factor TCF-1 (t cell factor 1, coded by gene *Tcf-7*) has been confirmed to promote the early fate commitment to the Tfh lineage over Th1 lineage during acute viral infection (32–34). Using the LCMV-Armstrong and influenza virus infection model, we (32) found that the expression level of TCF-1 was significantly enhanced in Tfh cells while greatly diminished in Th1 cells. And such divergent expression mode occurred as early as 2 days post infection. TCF-1 potently induced the expression of Bcl-6 but suppressed Blimp1 concomitantly, by directly binding to the *Bcl6* promoter region and *Prdm1* 5' regulatory region, respectively. Accordingly, virus-specific CD4+ T cells deficient in TCF-1 expression almost failed in Tfh differentiation. Notably, TCF-1 seems to specifically regulate Tfh cell differentiation in the context of viral infection, but dispensable for regulating Tfh differentiation during protein immunization (32, 33).

Apart from the master regulator Bcl-6, a network of several other transcription factors also participates in controlling the differentiation of Tfh cells during acute viral infection. For example, it has been confirmed that through two different but complementary mechanisms, the transcription factor KLF2 (Krüppel-like factor 2) functions to restrain Tfh cell generation. Lee et al. (35) found that KLF2 promotes the expression of the trafficking receptor S1PR1, the downregulation of which is essential for efficient Tfh cell differentiation. On the other hand, KLF2 favors the expression of several transcription factors that inhibit Tfh differentiation, such as Blimp1, Tbet, and GATA3. And KLF2 was also reported to suppress the transcription of *Cxcr5* by directly binding to its genomic region (36). Importantly, although Tbet is the master transcriptional regulator of Th1 cells, which were thought to inhibit Tfh cell differentiation, Tfh cells do exhibit medium to high levels of Tbet expression in the LCMV infection model (2). Recently, it has been reported that T-bet is virtually essential for the optimal expansion, proliferation, and maintenance of Tfh cells during acute viral infection (37). Besides, Fang et al. (38) demonstrated that at the early stage of CD4^+^ T cells response, the short-term expression of Tbet is critical for IFN-γ production in Th1-like Tfh cell subset. Additionally, transcription factors of the E-protein and Id families are well-appreciated for their role in T cell development. Shaw et al. (39) found that Tfh cells exhibited lower expression of Id2 than that of Th1 cells during acute viral infection and knockdown of Id2 via shRNA increased the frequency of Tfh cells. Furthermore, Th1 differentiation was significantly blocked by the deficiency of gene *Id2* during viral infection. Ogbe et al. (40) found that EGR2 (early growth response gene 2) and EGR3 play a vital role in directing the expression of *Bcl6* in Tfh cells. The differentiation of Tfh cells was impaired in *Egr2* and *Egr3* deficient mice post viral infection because of the defective expression of Bcl-6, resulting in a defective GC reaction and antibody production. Moreover, the overexpression of Bcl-6 in EGR2/3- deficient CD4^+^ T cells partially rescued the differentiation of Tfh cells and GC formation. Liu et al. (41) found that during influenza virus infection, the deletion of Ascl2 in T cells results in impaired Tfh-cell development and germinal center response. Besides, in protein immunization or other infection models, several other TFs have been confirmed to participate in the regulation of the fate commitment of Tfh cells. For example, c-Maf, IRF4, and Notch signaling pathway has been confirmed to promote Tfh differentiation while FOXO1 and FOXP1 inhibit Tfh fate commitment (21, 42–47). Besides networks mediated by transcriptional factors, other different signaling pathways also control the differentiation and function of Tfh cells. Tfh cell differentiation are closely associated with mTOR-mediated signaling pathways, which exert its effect by sensing and integrating environmental cues. During acute viral infection, the interleukin-2 (IL-2)-mTORC1 signaling axis orchestrates the reciprocal balance between Th1 and Tfh cell fates by promoting Th1 while inhibiting Tfh cell differentiation (20). In contrast, it is reported that mTORC2 was essential for Tfh cell differentiation (48, 49); specifically, mTORC2 mainly functions in the late stage of Tfh differentiation, promoting a Tfh transcriptional program and migratory ability toward B cell follicles (50).

Currently, however, our knowledge about Tfh cells is mainly derived from mouse models, although the gene expression pattern of mouse Tfh cells shares a high percentage of similarities with human Tfh, certain differences do exit between the two species. For instance, in mouse models, the ligand for CXCR5, CXCL13 is mainly expressed by stromal cells but not Tfh cells (6, 51). In humans, however, CXCL13 is primarily generated by Tfh cells, which may promote recruiting GC B cells to the light zone, where most Tfh cells and FDCs reside (52–54). Hence, further research is required for carefully profiling the differences between human and murine Tfh cells, which is critical for translating findings between the two species. Taken together, like Tfh cells developed in other scenarios, the fate commitment of virus-specific Tfh cells also follow a pathway involved a multistep and multifactorial process. Although we have gained a relatively detailed understanding of the ontology and differentiation of Tfh cells during viral infection, there are still important gaps in our knowledge of the Tfh cell differentiation and underlying mechanisms during viral infection both in mouse and humans. In particular, how extrinsic stimuli from APCs and intrinsic factors, such as epigenetic modifications, regulate anti-viral Tfh differentiation await further investigations.

## The Functional Maturation and Maintenance of Virus-Specific Tfh Cells

Currently, most licensed anti-viral vaccines protect vaccinated population by inducing long-lived neutralizing antibodies (55). Tfh cells play a critical role in helping B cells to differentiate into neutralizing-antibody-secreting plasma cells. Therefore, it is essential to understand the mechanisms by which Tfh cells co-opt B cell responses during viral infection, so as to effectively facilitate developing a novel vaccine or further improving the efficacies of licensed vaccines. Previous reviews have systematically summarized the functions of Tfh cells (6, 56), we herein mainly focus on the roles of these cells in the case of viral infection. After viral infection, SLAM-associated protein (SAP) expressed by Tfh cells is critical for the formation of germinal centers (57, 58), where Tfh cells facilitate the generation of long-lived memory B cells and plasma cells that produce virus-specific antibodies (57, 59). For example, CD4^+^ T cells have been confirmed to be essential for the generation of optimal antibody responses during infections with yellow fever virus (60), vaccinia virus (61), coronavirus (62), or vesicular stomatitis virus (VSV) (63). Collectively, Tfh cells play an important role in protective immunity to most, if not all, viruses.

In antiviral humoral immunity, Tfh cells help B cell activation and antibody production in the form of receptor ligand interactions and cytokine signaling. Firstly, Tfh cells highly express CD40 ligand (CD40L), whose interaction with CD40 expressed on B cells is vital to multiple stages and aspects of B cell response. Using LCMV, Pichinde virus, and VSV infection model, Borrow et al. (64) found that CD40L-deficient mice exhibit severely compromised humoral immune responses, supported by low antiviral antibody production, absence of germinal center and memory B cell formation. Consistently, CD40L/CD40 was also reported to be important for generating optimal humoral responses against HSV and influenza virus (65, 66). During LCMV, VSV, and influenza virus infection model, the expression of ICOS (inducible T cell co-stimulator) by Tfh cells has also been reported to be crucial for germinal center formation (6) and optimal induction of humoral responses (67). Other co-stimulatory molecules that promote the T-B conjugates, including SAP and SLAM family are also required for Tfh differentiation as well as Tfh function (6). It is important to appreciate that both defective Tfh cell number and damaged Tfh function can lead to impaired GC response. To more precisely evaluate the effector function of already differentiated Tfh cells during viral infection *in vivo*, our group combines *ERT2*^cre^ conditional knockout mice with mature Tfh cells adoptive transfer strategy to determine their “help” ability in promoting the formation of GC and plasma cells (32, 50). The expression of these B-cell helping molecules (CD40L and ICOS) in Tfh cells appears to be coordinated by Bcl-6 (68, 69), TCF-1 (32), and mTORC2 (50). Further studies are needed to determine the importance of additional molecular signals between Tfh cells and B cells in the production of protective antibody responses during viral infection.

## The Memory Formation of Virus-Specific Tfh Cells

Most of the virus-specific effector CD4^+^ T cells will die and only a small portion of them will survive and further differentiate into memory T cells after the elimination of a viral infection. The features of memory lymphocyte generally includes (1) antigen experienced (these cells have undergone antigen-driven expansion); (2) can survive for a long time (undergo homeostatic proliferation) in the absence of antigenic stimulation; (3) self-renewable by homeostatic proliferation; (4) rapidly recall their effector functions in response to re-challenge (70). Memory CD4^+^ T cells respond much faster than naive T cells, require less synergistic stimulation to respond to low antigen doses, and are more active when challenged by pathogens (71). Recent studies suggest both effector Tfh and Th1 cells can differentiate into memory cells.

Series of studies have clearly demonstrated the existence of memory Tfh cell in both mice and human (Figure 1) (2, 17, 72–75). These studies provide important insights into the characteristics of Tfh cells that can differentiate into long-lived memory-type cells which are endowed with capacity to reboost Tfh-specific effector functions when encountered with the same antigen (76). Meanwhile, considering the long persistence of GC reactions and antigen retention by FDC, it is important to appreciate that GC Tfh cells are not confined to one single GC. Once GC Tfh cells have differentiated and provided help to GC B cells, they can continually enter a different GC or exit GC and emigrate to neighboring follicles (77, 78), where no antigen was presented and Tfh cells acquires a less activated, less polarized phenotype. In this situation, by downregulating Bcl6 expression and upregulating IL-7Ra, Tfh cells gradually transit into a resting memory state (73, 78). In acute viral infection for instance, after the clearance of LCMV in mice, virus-specific CD4^+^ T cells that survived the contraction phase can be maintained for 60–150 days (2). Among these cells, CXCR5^+^Ly6c^lo^ resting CD4^+^ T cells shared similarities with effector Tfh cells, both phenotypically and transcriptionally, and can rapidly recall a secondary wave of effector Tfh response even in the absence of B cells, supporting that CXCR5^+^ memory cells have been imprinted with a Tfh-biased cell program (2). Cell markers that can clearly define memory Tfh cells including high expression of CXCR5, FR4 (79), CCR7, CD62L, and low expression of Bcl-6, ICOS, PD-1, and Ly6c (80). Currently, the differentiation pattern of memory Tfh cell remains controversial. An important question is whether the fate of memory Tfh cells is determined before or after effector phase. Meanwhile, given that Th1 vs. Tfh differentiation are regulated by strength and/or duration of TCR signaling (81, 82), which also influences memory CD4^+^ T cell differentiation (83), it is possible that Tfh effector population with different TCR strength varies in degrees of lineage commitment to CXCR5^+^ Tfh memory cells.

Besides, previous study indicated that memory Tfh cells are superior to naive T cells in helping B cells, and they promote faster B cell proliferation, higher antibody production and earlier class-switching reactions than naive CD4^+^ T cells (76). In many cases, the invasive virus can be quickly recognized and eradicated by pre-existing antibodies. But for viruses that bear high mutation rate (such as influenza virus and HIV), the most important point lies on faster antibody production, as the timely and efficient production of neutralizing antibodies targeting against new variants that escape from previously produced antibodies will be of great significance. Memory Tfh cells maintain a substantial level of CD40L (84) and are retained in the draining lymph nodes for more than 6 months (85). Besides, during several types of viral infections, such as Ebola virus (86), WNV (87), and influenza virus (88), memory Tfh cells can generate higher levels of cytokines as compared with those of naive T cells, which are likely to induce a more-potent B cell responses and to dictate the isotype of antibodies, which play key roles in the antibody responses specific for aforementioned viruses. Nonetheless, we currently know much less as to the molecular mechanisms underlying memory Tfh differentiation than those discovered with effector Tfh cells.

## The Differentiation of Virus-Specific Tfh Cells During Chronic Viral Infection

We know far less about how chronic viral infections affect CD4^+^ T cell responses than we do about CD8^+^ T cell exhaustion. However, increasing attention has been paid to the impact of persistent viral infection on the function of CD4^+^ T cells and the importance of CD4^+^ T cells in chronic viral infection. Compared with acute viral infection, virus-specific CD4^+^ T cells in LCMV clone 13 persistent infection model exhibit deficiency in production of Th1-type effector cytokines and fail to function optimally following viral re-challenge (89). The loss of CD4^+^ T cell's ability to respond to persistent antigens may be due to high levels of antigens at the priming stage (90) and appears not to be regulated by the changes in APCs caused by chronic viral pathogens (89).

Similar to CD8^+^ T cell exhaustion in chronic infection, the virus-specific CD4^+^ T cell response has been altered profoundly as infection persists. The most significant phenotype of CD4^+^ T cell response during chronic viral infection is a defect in Th1, while increasing in Tfh response (Figure 1). And both in mouse and human chronic viral infections, the frequency of CXCR5^+^CD4^+^ T cells in spleen accumulates gradually, reaching approximately 60~70% of the viral-specific CD4^+^ T cells by day 30, whereas which were relatively lower at a frequency of 40~50% during Arm infection (91). The increased Tfh differentiation was accompanied by a loss in Th1, including decreased proliferative potential and cytokine production (89). Thereby, to some extent, the immune system promotes antibody responses, which bear less immune-pathological risk compared to cytotoxic and pro-inflammatory T cell responses. Moreover, transcriptional profiling of viral-specific CD4^+^ T cells in LCMV clone 13 infection identified a loss in Th1 transcriptional signatures, as well as an enrichment of Tfh-associated transcripts (92). Upregulated CXCR5, Bcl-6, ICOS, OX40, and IL-21 expression suggest an enhanced Tfh-like CD4^+^ T cells phenotype (91). Very importantly, these additional Tfh-like CD4^+^ T cells are proven to have the ability to help B cells through *in vitro* culture, suggesting that they are equipped with some key signatures of conceptual Tfh cells and remain suboptimal functions, such as the ability to facilitate coordinate B cell response and production of antibody (91, 93). Although we have not yet got a comprehensive understanding of the biased differentiation of Tfh cells in chronic viral infection, previous research proved that type I IFN signaling may be an important mediator involved in the shift from Th1 to Tfh cells (94–97). Several groups also demonstrated the skewed differentiation toward Tfh cells during chronic LCMV infection is firmly related to cytokines (such as IL-6/ IL-27) signaling through the IL-6 family receptor pathway (98, 99). Recently, Raju et al. (100) found that the deficiency of the signaling adaptor CD2AP (CD2-associated protein) promotes CD4^+^ T cell differentiation toward Tfh lineage during chronic LCMV infection, leading to better control of viral infection by enhanced GC response. They demonstrated that the strengthened Tfh differentiation is associated with extended duration of TCR signaling and enhanced cytokine production of CD2AP-deficient CD4^+^ T cells specifically under Th1 conditions. To be noted, the increased CXCR5 level may also contributed by another CD4^+^ T cell subpopulation, Tfr cells (101, 102), coincide with upregulated Foxp3 expression during chronic infection (103). Although the function of Tfr cells is incompletely understood, especially in chronic infection, the increased Tfr differentiation suggest an active follicular program in chronic infection. And this follicular program may be not only confined to CD4^+^ T cell lineage, confirmed by newly identified CXCR5^+^CD8^+^ T cells described both in mice and human chronic viral infection (104–106).

Although viral persistence redirects a shift in Tfh differentiation, it is not clear to what extent the function of Tfh cells generated during chronic viral infection gets changed. IL-21, canonical Tfh cytokine important for CD8^+^ T cell function in chronic viral infection (107–110), is increased within Tfh population. However, for humoral immunity, it seems that B cell could not get optimal help from increased Tfh in chronic LCMV infection. Firstly, the generation of neutralizing antibodies are impaired and delayed, whereas, non-neutralizing antibodies to LCMV increased considerably (111). Secondly, persisting viral infections can lead to polyclonal hypergammaglobulinemia and antibody-mediated autoimmunity, results of non-specific B cell activation by Tfh cells (112, 113). The delayed production of neutralizing antibodies and high production of poor quality antibodies indicate that the interaction between Tfh and B cells in chronic LCMV infection is dysregulated, leading to a suboptimal ability of Tfh to help B cells producing high-affinity antibodies.

As has been observed in LCMV, HIV, and SIV infections also have been reported to have increased frequency of CXCR5^+^CD4^+^ T cells (114, 115). Besides, compared with uninfected healthy donors, the transcription characteristics of Tfh cells in the SIV infection model were changed. It has been confirmed that the transcriptional signature of Tfh cells derived from SIV-infection models gets remarkably altered compared to those from healthy donors (116). The underlying mechanisms initiating and promoting a Tfh-like program in HIV infection still remain unsolved and the relationship between HIV infection and Tfh differentiation is complicated. During HIV or SIV infections, Tfh cells seem to act in a bilateral manner, both immunological and immunopathogenical: Firstly, Tfh cells are appreciated as an important cellular reservoir of replication-competent HIV virus, contributed by its special phenotype and follicular localization. It is demonstrated that Tfh cells located in B-cell follicles are preferentially targeted by HIV virus to form both long-term latent infection and the enduring generation of virulent particles (110, 117), and the anatomical separation of latently infected Tfh cells might represent a major barrier for HIV-specific CD8^+^ T cells, which are normally excluded from B-cell follicles, to effectively eradicate HIV infection (118–121). Secondly, Tfh is closely involved in developing antibody-based vaccines for HIV-1 infection, because functional Tfh-B cell interactions are key to production of effective antibodies in vaccination (16, 122). Consistent with mouse chronic infection models, Tfh cells do not provide adequate help to B cells even though these cells are expanded in HIV-infected individuals (increased Tfh frequency dose not result in better B cell response). Instead, similar to mouse chronic infection of LCMV, abnormal B cell activation and hyper-gammaglobulinemia were observed in HIV-1 infection (112, 115, 123), which suggests the dysregulation of Tfh cell-mediated B cell help and disturbed Tfh-B cell interactions. Specifically, data from mass cytometry combine with TCR sequencing confirmed that compared with healthy individuals, Tfh cells in the lymph nodes of HIV+ individuals secreted interleukin-21 but were functionally and clonally restricted and this correlated with impaired isotype switching of B cells in the lymph nodes (124). Given the close relationship with Tfh from lymphoid tissues, circulating or peripheral Tfh cells have also been confirmed to be critical in HIV infection (16, 22, 125). He et al. (18) demonstrated that circulating CXCR5^+^CD4^+^ T cells are generated in a SAP independent manner (before they migrate to GC), and CCR7^lo^PD-1^hi^ subset correlated with Tfh cell activity, providing a biomarker to monitor protective humoral immune responses during infection or vaccination. In a related study, combining cytokine production, functional properties as well as gene expression profile, Locci et al. (17) identified pTfh cells related to germinal center Tfh cells as resting CD45RO^+^PD-1^+^CXCR5^+^CXCR3^−^CD4^+^ T cells. And they confirmed that the frequency of this population positively correlates with the titers of HIV-specific broadly neutralizing antibodies in a large cohort of HIV-infected patients. Schultz et al. (15) found that during HIV infection, peripheral IL-21^+^ CD4^+^ T cells show similarities with lymphoid tissue-resident Tfh cells phenotypically, transcriptionally, and functionally. And they also found that the numbers of HIV-specific IL-21-expressing pTfh cell increased and their number positively correlated with antibody production in the ALVAC priming, AIDSVAX boosting immunization strategy used in the RV144 trial (the only HIV vaccine to demonstrate some signs of efficacy among human patients) when compared with the non-protective DNA prime-Ad5 boosting vaccine trial. Given that the timely development of high-affinity antibodies is central to the prevention and eradication of viral infection (126), further work is needed to understand the detailed mechanism underlying Tfh dysfunction during persistent viral infections.

The key feature of CD8^+^ T cell exhaustion is upregulated expression of co-inhibitory receptors, such as PD-1, Tim3, 2B4. Although CD4^+^ T cells sustained the expression of a sets of co-inhibitors (127), however, the specific inhibitory receptors upregulated and the degree of expression between CD4^+^ and CD8^+^ T cells differed remarkably (92, 127). For example, the expression of 2B4 is biased toward exhausted CD8^+^ T cells, while PD-1, CTLA4 are preferentially expressed in CD4^+^ T cells, particularly in Tfh cells (92). Several groups (128) found that functionally impaired CD4^+^ T cells derived from HIV patients exhibit significant enhancement in proliferative potential after treatment targeting on CTLA-4 (129, 130), TIM3 (131) or PD-1 signaling blockade (132) *in vitro*. And the effector function of CD8^+^ T cells can be rescued through enhancement of CD4^+^ T cell response during chronic infection with LCMV (133, 134). These findings and others (135, 136) shed new lights on the design of vaccines against chronic viral infections. For Tfh cells, physiologically, PD-1 is assigned to provide inhibitory signals to GC Tfh cells, preventing excess cell proliferation during GC reaction (21). Good-Jacobson et al. (137) demonstrated that upon immunization, the deficiency of PD-1 or PD-1 ligands (PD-L1/PD-L2) results in higher frequency of Tfh cells. Whereas, they also found that the quality of Tfh cells is dramatically impaired by diminishing their capacity to synthesize important cytokines (such as IL-4/IL-21) while not promoting the development of an alternatively polarized T cell type. These results suggest a complex but critical role of PD-1 in Tfh cells. However, despite the above findings, the role of PD-1 in Tfh cells during chronic viral infection remains unclear. Whether the expression of PD-1 on Tfh cells equals exhaustion or whether this is part of their normal regulation and functional differentiation during persistent infection have not yet been fully discovered.

Most of the aforementioned knowledge about CD4^+^ T cell response during chronic viral infection has been obtained from studies in which animals are infected with a single virus. While valuable for identification of basic principles, this is not reflective of human biology, since human beings undergo repeated viral infections throughout their life span, most notably, multiple herpesviruses. The γ-herpesviruses (Gammaherpesviruses), including EBV (Epstein-Barr virus) and KSHV (Kaposi's sarcoma-associated herpesvirus), are associated with lymphoproliferative diseases and lymphomas and with the majority establishing latency in B lymphocytes (138). In mouse models, intranasal infection of mice with the murine γ-herpesvirus (MHV-68, shares biological and genetic homology with EBV) results in an acute lytic infection in the lung, followed by the establishment of lifelong latency in memory B cells, dendritic cells, and macrophages (104, 139–141). Barton et al. (142) demonstrated that both the proportion and total number of IFNγ^+^, TNFα^+^, and IL-2^+^ CD4^+^ T cells was increased in mice infected with MHV68 followed with LCMV-Armstrong re-challenge compared to that in mice solely infected with LCMV on day 8 post infection. This result reminded us that MHV68 latency may provide micro-environment in which effector CD4^+^ T cell responses get enhanced during subsequent infection. Another study confirmed that signals from Tfh cell is critical for B cell latency during MHV68 infection. They found that the absence of these signals lead to a significant reduction in the number of MHV68 latently infected B cells (143). However, whether Tfh cells are selectively up-regulated during MHV68 chronic infection has not yet been fully illustrated. Apart from its fundamental role in supporting B cell latency in MHV68 infection, CD4^+^ T cells may also control MHV68 replication in a CD8^+^ T cell dependent or independent manner (138, 144). Recently, several groups (104, 145) identified a specialized group of cytotoxic T cells that expressed high level of the chemokine receptor CXCR5 (Tfc, Follicular cytotoxic T cells), which selectively entered B cell follicles and eradicated infected Tfh cells and B cells during HIV/SIV or EBV infection, respectively. Given that Tfh and Tfc cells have a similar histological location, it will be of interests to determine whether these two subsets have interaction or crosstalk during chronic viral infection.

Collectively speaking, further dissection of unique molecular mechanisms underlying differentiation and functionality of Tfh cells in chronic viral infection will provide opportunities for harnessing this population to prevent and treat chronic viral infection.

## Perspective

Currently, the transcriptional regulation of the ontogeny and development of Tfh cell has been extensively investigated. However, the field just starts to dissect the complexities of cellular metabolism within Tfh cell as well as its epigenetic signatures, particularly, in the scenario of viral infections. It is well-acknowledged that the differentiation of Tfh cells is accompanied by unique metabolic alterations required to meet their cellular bioenergetic demands. During acute viral infection, Tfh cells exhibited a relatively quiescent metabolic state when compared to Th1 lineage, characterized by reduced glucose uptake and mitochondrial respiration, as well as lowered maximal respiratory capacity and extracellular acidification. However, despite Tfh cells showing reduced metabolic capacity, they still require glycolysis as well as oxidative phosphorylation to provide sufficient energy and substrates for their specific function (20). Zeng et al. (49) found that mTOR, combining metabolic signals and transcriptional activity, plays as a central control station in Tfh differentiation. Activated by costimulatory molecule ICOS, mTOR acts to drive glycolysis and lipogenesis and subsequently promotes Tfh cell responses during acute viral infection. Given that GC-Tfh cells have a different localization compared to outside Tfh cells. They may have distinct metabolic features influenced by unique cellular and nutritional contact within each microenvironment. Moreover, it is possible that memory Tfh cells differs from effector Tfh cells in metabolism as described in effector vs. memory CD8^+^ T cells. Whether and how cellular metabolism influence the formation of memory Tfh cells still need further investigation.

Besides metabolic issues, the differentiation of Tfh cells as well as other CD4^+^ T helper (Th) cells are firmly correlated with specific epigenetic modifications (146, 147). By generating T cell-specific UTX (ubiquitously transcribed tetratricopeptide repeat, X chromosome) deficient mice, Cook et al. (148) found that during chronic but not acute, virus infection, Tfh differentiation were significantly impaired in UTX deficient mice, which in turn leads to suboptimal formation of germinal center and production of virus-specific IgG. Mechanistically, the absence of UTX leads to the upregulation of H3K27 methylation which further results in decreased expression of IL-6R alpha and other Tfh lineage-related genes. Nishizawa et al. (149) demonstrated that Bcl-6 is highly expressed in angioimmunoblastic T-cell lymphoma (AITL) and peripheral T-cell lymphomas (PTCL) containing tumor cells with Tfh features. In their research, hypermethylation of the *Bcl6* locus followed by Bcl-6 upregulation, combined with *TET*2 mutations, was thought to be the key event for lymphoma development which may result in biased Tfh differentiation and eventually contribute to AITL/PTCL development in patients. Apart from these achievements, there are still important gaps in our knowledge of the epigenetic features of Tfh cells. Further studies will be required to draw a comprehensive epigenetic landscape of Tfh cells and identify potential candidate chromatin modifiers that participate in Tfh development. Understanding these issues and dissecting the underlying regulatory mechanisms will advance our knowledge of Tfh cells and shed lights on designing new strategies against those diseases associated with Tfh abnormalities.

## Ethics Statement

All of our studies were specifically reviewed and approved by the Institutional Animal Care and Use Committees of the Third Military Medical University.

## Author Contributions

QH, JH, and JT wrote and edited the manuscript with LX and LY.

### Conflict of Interest Statement

The authors declare that the research was conducted in the absence of any commercial or financial relationships that could be construed as a potential conflict of interest.
